# Rhoifolin Ameliorates Osteoarthritis *via* Regulating Autophagy

**DOI:** 10.3389/fphar.2021.661072

**Published:** 2021-05-28

**Authors:** Jiyuan Yan, Bowei Ni, Gaohong Sheng, Yingchi Zhang, Yifan Xiao, Yongzhuang Ma, Hao Li, Hua Wu, Chang Tu

**Affiliations:** ^1^ Department of Orthopedics, Tongji Hospital, Tongji Medical College, Huazhong University of Science and Technology, Wuhan, China; ^2^ Department of Orthopedics, Renmin Hospital of Wuhan University, Wuhan, China; ^3^ Department of Pathology and Pathophysiology, Medical College, Jianghan University, Wuhan, China; ^4^ Department of Traumatology, Tongji Hospital, Tongji Medical College, Huazhong University of Science and Technology, Wuhan, China; ^5^ Department of Orthopedics, Shanxi Bethune Hospital, Taiyuan, China

**Keywords:** rhoifolin, osteoarthritis, autophagy, 3-methyladenine, MAPK, PI3K/AKT/mTOR

## Abstract

Osteoarthritis (OA) is a common age-related joint disease. Its development has been generally thought to be associated with inflammation and autophagy. Rhoifolin (ROF), a flavanone extracted from *Rhus succedanea*, has exhibited prominent anti-oxidative and anti-inflammatory properties in several diseases. However the exact role of ROF in OA remains unclear. Here, we investigated the therapeutic effects as well as the underlying mechanism of ROF on rat OA. Our results indicated that ROF could significantly alleviate the IL-1β–induced inflammatory responses, cartilage degradation, and autophagy downregulation in rat chondrocytes. Moreover, administration of autophagy inhibitor 3-methyladenine (3-MA) could reverse the anti-inflammatory and anti-cartilage degradation effects of ROF. Furthermore, P38/JNK and PI3K/AKT/mTOR signal pathways were involved in the protective effects of ROF. *In vivo*, intra-articular injection of ROF could notably ameliorate the cartilage damage in rat OA model. In conclusion, our work elucidated that ROF ameliorated rat OA *via* regulating autophagy, indicating the potential role of ROF in OA therapy.

## Introduction

Osteoarthritis, featured with cartilage loss, joint pain, and physical disability, has attracted worldwide attention in recent years (Kontio et al., 2020). As an age-related joint disease, OA afflicts over 240 million people globally. Furthermore, the prevalence of OA continues to rise since the mid-twentieth century (Nelson 2018; Mandl 2019). Currently, most therapeutic strategies targeted for OA focus on relieving the symptom rather than reversing the disease progression. Therefore, patients at the late stage of OA have to accept joint replacement without better choices (Glyn-Jones et al., 2015).

Chronic inflammatory responses and cartilage metabolic imbalances are vital to the progression of OA (Appleton, 2018). Excessive release of proinflammatory cytokine interleukin-1β (IL-1β) was found in the synovial fluid of OA patient (Pelletier et al., 2001). Previous study indicated that IL-1β could induce the production of matrix metallo-proteinases (MMPs) and aggrecanase-2 (ADAMTS5), which resulted in the loss of cartilage matrix (Tu et al., 2019). Moreover, elevated level of IL-1β had been shown to exacerbate the inflammatory responses via overproducing the inflammatory mediators including cyclooxygenase-2 (COX-2) and inducible nitric oxide synthase (iNOS) (Tu et al., 2019). Autophagy is a highly conserved catabolic process which is essential for maintaining cell homeostasis (He and Klionsky 2009). In OA pathological progression, autophagy induction could promote chondrocyte survival and cartilage matrix synthesis (Carames et al., 2010). Besides, intra-articular injection of autophagy inhibitor ameliorated the cartilage degradation in mouse OA model (Takayama et al., 2014). Furthermore, it is reported that IL-1β could significantly induce the autophagy downregulation in chondrocytes (Wang et al., 2019). Therefore, strategies targeting IL-1β may lead to new ideas in OA treatment.

Due to mild side effects and definite efficacy, more and more herb medicines have been accepted by OA patients (Li et al., 2017). Rhoifolin, a bioactive product first extracted from *Rhus succedanea*, has proved its anti-oxidative and anti-inflammatory properties in several diseases (Fang et al., 2020; Gandhi et al., 2020; Peng et al., 2020). *In vitro*, ROF attenuated osteoclasts-stimulated ostolysis *via* suppressing MAPK and NFκB signal pathways, indicating the possible mechanism of its action (Liao et al., 2019). However, the detailed role of ROF in OA remains unclear. In this study, we aim to clarify therapeutic potential of ROF in OA and in the underlying mechanism. We expect to explore a new way in OA treatment.

## Materials and Methods

### Ethics Approval

This study was conducted in strict accordance with the Guidelines of Animal Care and Use Committee for Teaching and Research, Tongji Medical College, Huazhong University of Science and Technology. The experimental procedures were approved by the Institutional Animal Care and Use Committee, Tongji Medical College, Huazhong University of Science and Technology. All efforts were made to reduce animal suffering.

### Reagents

Rhoifolin (ROF, #PHL83302) was procured from Sigma Aldrich (St. Louis, MO, United States). ROF was dissolved in DMSO and all experiment groups were treated with equal volume of DMSO. Recombinant rat IL-1β was purchased from R and D systems (Minneapolis, MN, United States). Fetal bovine serum (FBS) and Dulbecco’s Modified Eagle’s Medium F12 (DMEM/F12) were acquired from Gibco (NY, United States). Antibodies specific for MMP13 (#ab219620) and iNOS (#ab136918) were provided by Abcam (Cambridge, MA, United States). Antibody against Collagen Ⅱ (#15943-1-AP) was obtained from Proteintech Group (Wuhan, Hubei, China). Antibodies against COX-2 (#12882), ATG5(#12994), ATG12 (#4180), Beclin-1 (#3495), P62-sequestosome (#39749), LC3 Ⅰ/Ⅱ (#12741), P-PI3K(#4228), PI3K(#4249), P-AKT (#4060), AKT(#4691), P-mTOR (#5536), mTOR (#2983), P-JNK (#9255), JNK (#9258), P-P38 (#4511), P38 (#8690), P-ERK (#4370), and ERK (#4695) were supplied by Cell Signaling Technology (Beverly, MA, United States). Antibodies specific for ADAMTS5 (#BA3020) and GAPDH (#BM3876) were procured from Boster (Wuhan, Hubei, China).

### Cell Culture

Chondrocytes were harvested from 5 days-old Sprague–Dawley (SD) rats as reported previously (Ma et al., 2019; Tu et al., 2019). Briefly, the cartilage obtained from the knee joints was first sliced into small pieces and digested with 0.25% trypsin–EDTA for 30 min. Then, the cartilage fragments were fully digested with 0.25% collagenase Ⅱ overnight. Subsequently, the cell suspension was centrifuged with a speed of 1,500 rpm for 5 min to obtain primary chondrocytes. Cells were finally cultured in DMEM/F12 supplemented with 10% FBS and 1% penicillin/streptomycin solution at 37°C with 5% CO_2_. Chondrocytes of second or third passages were used in following experiments.

### Cell Viability

Cell Counting Kit-8 (CCK-8, #AR1160, Boster) assay was employed to explore the cell viability. Concisely, rat chondrocytes were seeded onto 96-well plates at a density of 1 × 10^4^ cells per well. After adhesion, the cells were treated with different concentrations of ROF (0, 5, 10, and 20 μM) alone or combined with IL-1β (10 ng/ml) for 24 h. Next, 100 μL culture medium supplemented with 10 μL CCK-8 reagent was added to each well. After 1 h incubation at room temperature, the absorbance at 450 nm of each well was detected by a microplate reader (Bio-Rad, Richmond, CA, United States).

### Western Blot

Rat chondrocytes were collected and washed with phosphate buffered saline (PBS). Next, the cells were lysed with RIPA buffer containing 1% protease/phosphatase inhibitor cocktail (Boster). The protein concentration of isolated cell lysis solution was determined by bicinchoninic acid (BCA) kit. Afterward, 25 μg protein samples were separated on 8–12% SDS–polyacrylamide gels and transferred to PVDF membranes using a Bio-Rad system. The membranes were blocked with 5% BSA for 1 h at room temperature and then incubated with primary antibodies overnight at 4°C. Subsequently, the membranes were washed with TBST and incubated with corresponding secondary antibodies for 1 h at room temperature. Finally, enhanced ECL kit (Thermo Fisher Scientific, United States) was used to visualize the blots. The relative protein expression was calculated by ImageJ software compared to internal control.

### Tandem GFP-RFP-LC3 Adenovirus Transfection

For autophagy flux evaluation, rat chondrocytes were transfected with tandem GFP-RFP-LC3 adenovirus transfection vectors (HanBio Technology, China). Autophagosomes (shown in green) and autolysosomes (shown in red) were observed with a nanoscale laser scanning confocal microscope system (Nikon, NY, United States).

### Transmission Electron Microscopy

Rat chondrocytes were trypsinized, centrifuged and blocked in 10% BSA. Then cells were fixed using 2.5% glutaraldehyde overnight at 4°C. Subsequently, the cells were washed with PBS and fixed with 1% osmium tetroxide for 1 h. Next, the cells were washed with distilled water and stained using 2% uranyl acetate for 1 h. After dehydration, the samples were mixed with resin and propylene oxide (1:1) for 2 h and transferred into pure resin overnight. Finally, the samples were embedded, sectioned, and stained with uranyl acetate and lead citrate. A TECNAI G20 transmission electron microscopy (TEM) was employed to observe the autophagosomes in cells.

### Rat Osteoarthritis Model

Eighteen 8-week-old male SD rats (weight 280–320 g) were obtained from the Laboratory Animal Center of Tongji Hospital. The rat OA model was built by anterior cruciate ligament transaction (ACL-T) and medial meniscus destabilization as described before (Appleton et al., 2007). All rats accepted the surgery on the right knee and were randomly divided into three groups. For the ROF group (*n* = 6), rats were performed the operation and intra-articular injection of 20 μM ROF weekly. For the sham group (*n* = 6), rats accepted sham surgery without ACL-T or meniscus destabilization and were treated with equal volume of saline. For the OA group (*n* = 6), rats were performed the operation and intra-articular injection of equal volume of saline. 8 weeks postsurgery, all rats were sacrificed and the samples were fixed with 4% paraformaldehyde for further study.

### Histological Evaluation

Fixed samples were decalcified in 10% EDTA solution for 1 month and embedded in paraffin. Then the whole knee joints were cut into 5 μm-thick sections coronally, and stained with H and E and Safranin-O-Fast green. Further immunohistological staining was performed using antibodies against Collagen Ⅱ, MMP13, and LC3B. Furthermore, the Osteoarthritis Research Society International (OARST) scoring system was employed to evaluate the histological changes.

### Statistical Analysis

Data are exhibited as mean ± standard deviation. All data were analyzed using one-way analysis of variance (ANOVA) followed by Turkey’s post hoc test. Comparisons were considered significant for *p <* 0.05. All experiments were repeated at least three times.

## Results

### Effects of Rhoifolin on Cell Viability

**FIGURE 1:**
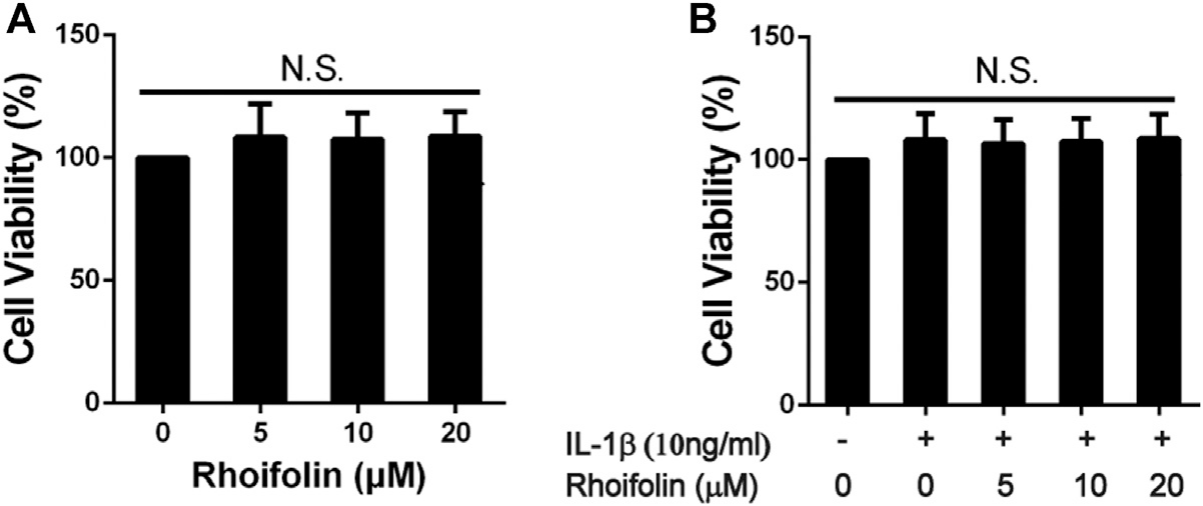
Effects of ROF on Cell Viability. **(A)** Rat chondrocytes were exposed to ROF (5, 10, 20 μM) alone or **(B)** with IL-1β (10 ng/ml) for 24 h and cell viability was assessed by CCK-8 assay. N.S. indicated no significance.

CCK-8 assay was performed to evaluate the cytotoxic effects of ROF on rat chondrocytes. As shown in Figure 1, after 24 h reaction, ROF at the concentrations of 5, 10, and 20 μM had no toxic effects on chondrocytes with or without IL-1β treatment (10 ng/ml).

### Rhoifolin Alleviates IL-1β–Induced Inflammatory Responses and Cartilage Degradation

**FIGURE 2:**
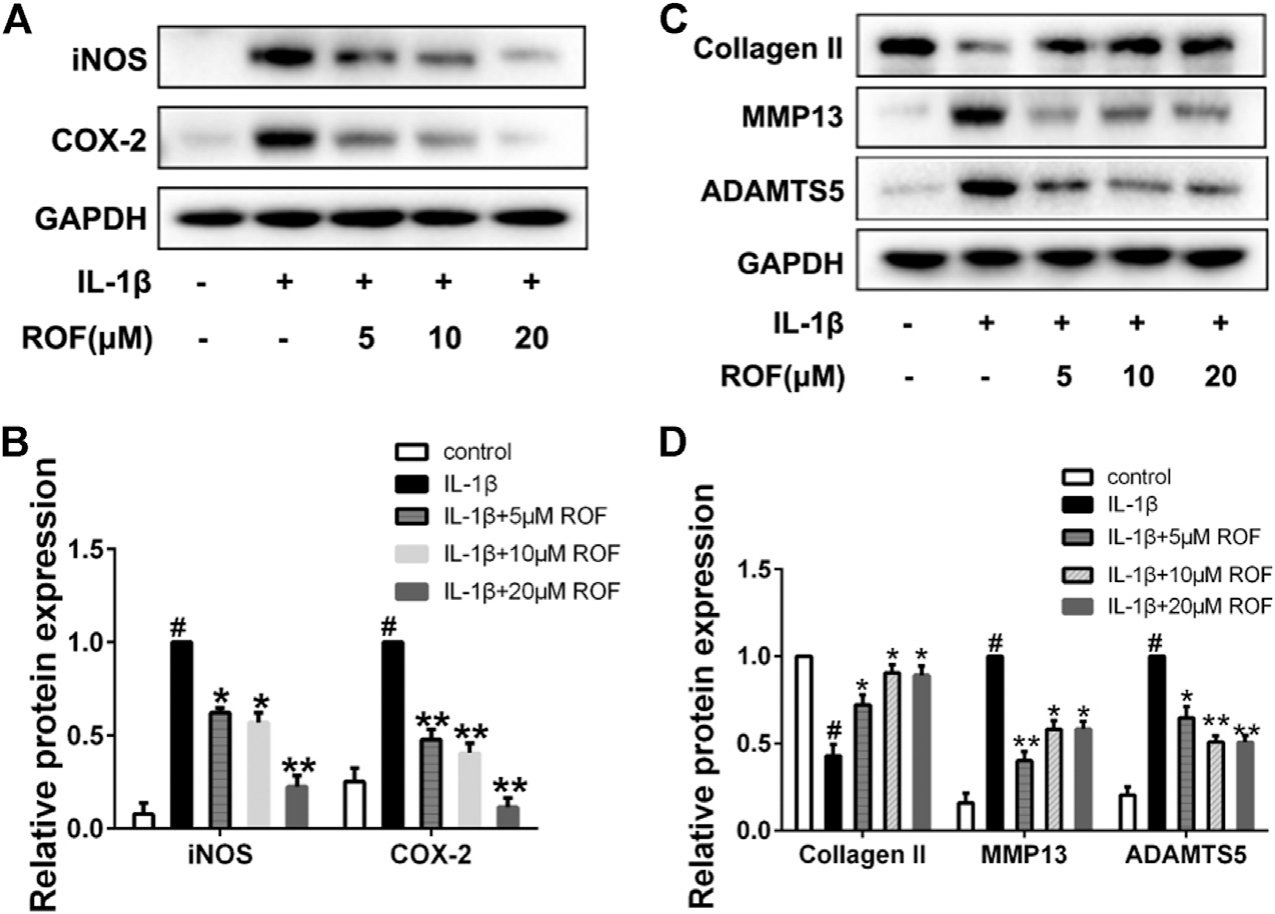
ROF inhibits IL-1β–induced inflammatory responses and cartilage degradation in chondrocytes. Cells were treated with different concentrations of ROF in the presence or absence of IL-1β (10 ng/ml) for 24 h. **(A)** Western blots and **(B)** quantitative analysis of iNOS and COX-2. **(C)** Western blots and **(D)** quantitative analysis of collagen Ⅱ, MMP13, and ADAMTS5. GAPDH was employed as the loading control (*n* = 3). #*p* < 0.05 vs. control group; *p < 0.05 and **p < 0.01 vs. IL-1β group.

To confirm the protective effects of ROF on the IL-1β–induced inflammatory responses and cartilage degradation in rat chondrocytes, Western blotting was performed. As exhibited in Figures 2A,B, IL-1β could significantly increase the expression of inflammatory cytokines, including iNOS and COX-2, while administration of different concentrations of ROF could alleviate this process. Moreover, the expression of collagen Ⅱ, MMP13, and ADAMTS5 was used to evaluate the cartilage degradation. As shown in Figures 2C,D, IL-1β could notably induce the upregulation of MMP13 and ADMTS5 and the downregulation of collagen Ⅱ. However, ROF could reverse this change.

### Rhoifolin Attenuates IL-1β–Induced Autophagy Downregulation in Chondrocytes

**FIGURE 3:**
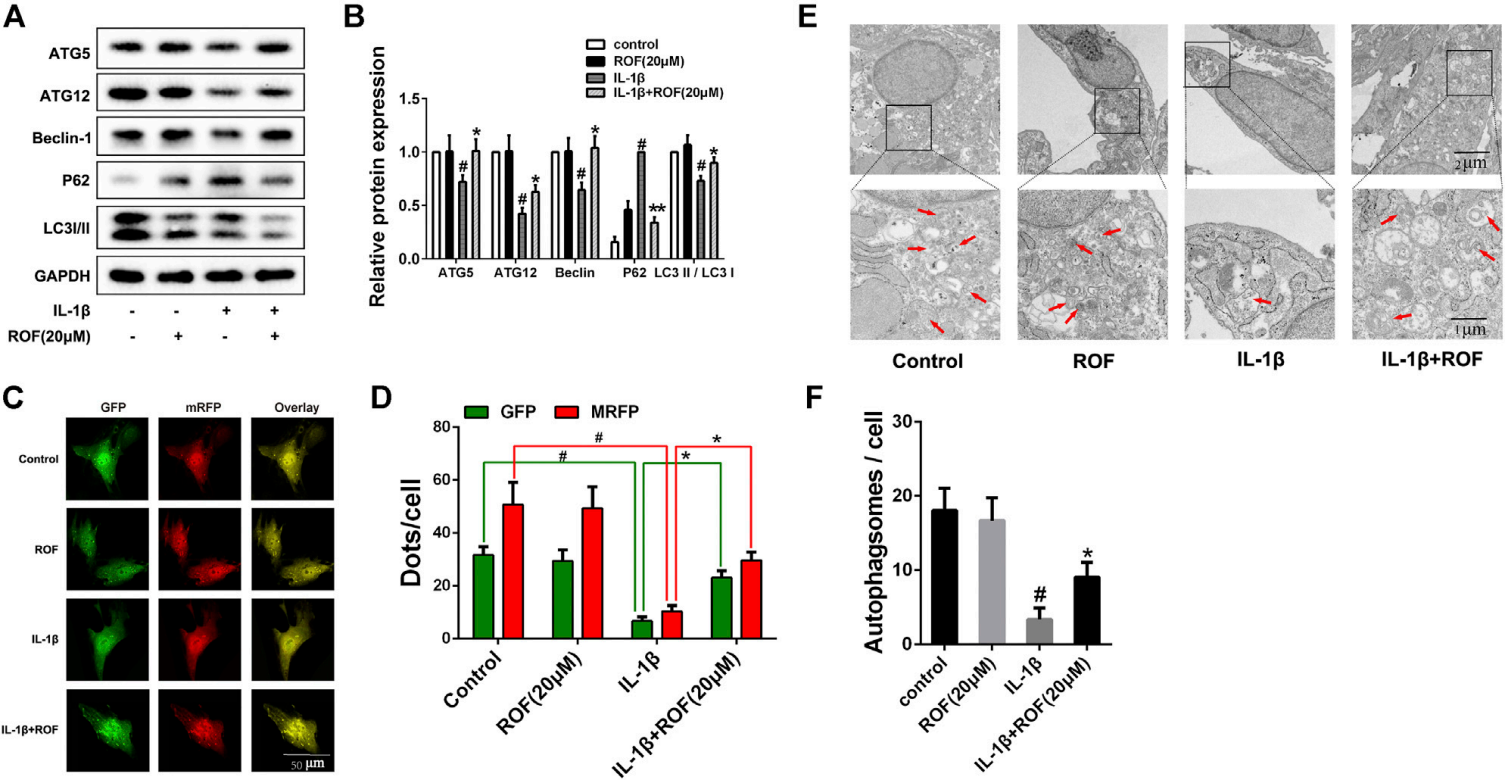
ROF suppresses IL-1β–induced autophagy downregulation in chondrocytes. **(A)** Western blots and **(B)** quantitative analysis of ATG5, ATG12, Beclin-1, P62-sequestosome and LC3 Ⅱ/LC3 Ⅰ in each group. **(C)** Fluorescence microscopy and **(D)** quantitative analysis of cells transfected with tandem GFP-RFP-LC3 adenovirus in each group. **(E)** TEM images and **(F)** quantitative analysis of autophagosomes in each group. Red arrow indicated autophagosome. GAPDH was employed as the internal control (*n* = 3). #*p* < 0.05 vs. control group; *p < 0.05 vs. IL-1β group.

A previous study indicated that autophagy played an important role in OA development. We next explored the effects of ROF on autophagy in IL-1β–treated rat chondrocytes. As shown in Figures 3A,B, IL-1β treatment significantly decreased the expression of ATG5, ATG12, Beclin-1, and the ratio of LC3 Ⅱ/LC3 Ⅰ, while increased the expression of P62-sequestosome in rat chondrocytes. However, administration of 20 μM ROF could markedly reverse this change. The tandem GFP-RFP-LC3 adenovirus transfection was further used to observe the autophagosome formation. As exhibited in Figures 3C,D, the number of autophagosomes (green puncta) and autophagolysosomes (red puncta) in chondrocytes decreased significantly after IL-1β treatment. However, when treated with 20 μM ROF, more autophagosomes and autophagolysosomes were observed in chondrocytes. Moreover, transmission electron microscopy (TEM) was used to further observe the autophagosomes in each group. As shown in Figures 3E,F, the ROF treatment could significantly alleviate the IL-1β–induced autophagosomes decrease in rat chondrocytes.

#### Methyladenine Reverses the Anti-Inflammatory and Anti-Cartilage Degradation Effects of Rhoifolin in Chondrocytes

**FIGURE 4:**
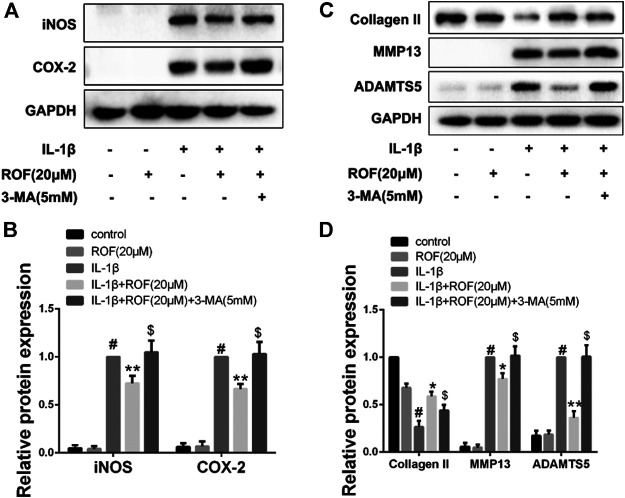
Autophagy inhibitor 3-MA attenuates the anti-inflammatory and anti-cartilage degradation effects of ROF in chondrocytes. **(A)** Western blots and **(B)** quantitative analysis of iNOS and COX-2 in each group. **(C)** Western blots and **(D)** quantitative analysis of collagen Ⅱ, MMP13, and ADAMTS5 in each group. GAPDH was employed as the internal control (*n* = 3). #*p* < 0.05 vs. control group; *p < 0.05 and **p < 0.01 vs. IL-1β group;^$^ *p* < 0.05 vs. IL-1β + ROF (20 μM) group.

To elucidate whether ROF exerts anti-inflammatory and anti-cartilage degradation effects by regulating autophagy, autophagy inhibitor 3-methyladenine (3-MA) was employed. As shown in Figure 4, ROF could effectively ameliorate the IL-1β–induced inflammatory response and cartilage matrix degradation in chondrocytes. However, when 3-MA was added simultaneously, the protective effects of ROF were blocked. The results suggested that ROF may function via promoting autophagy in IL-1β–treated rat chondrocytes.

### Effects of Rhoifolin on Interleukin-1β–Induced MAPK and PI3K/AKT/mTOR Signal Activation in Chondrocytes

**FIGURE 5:**
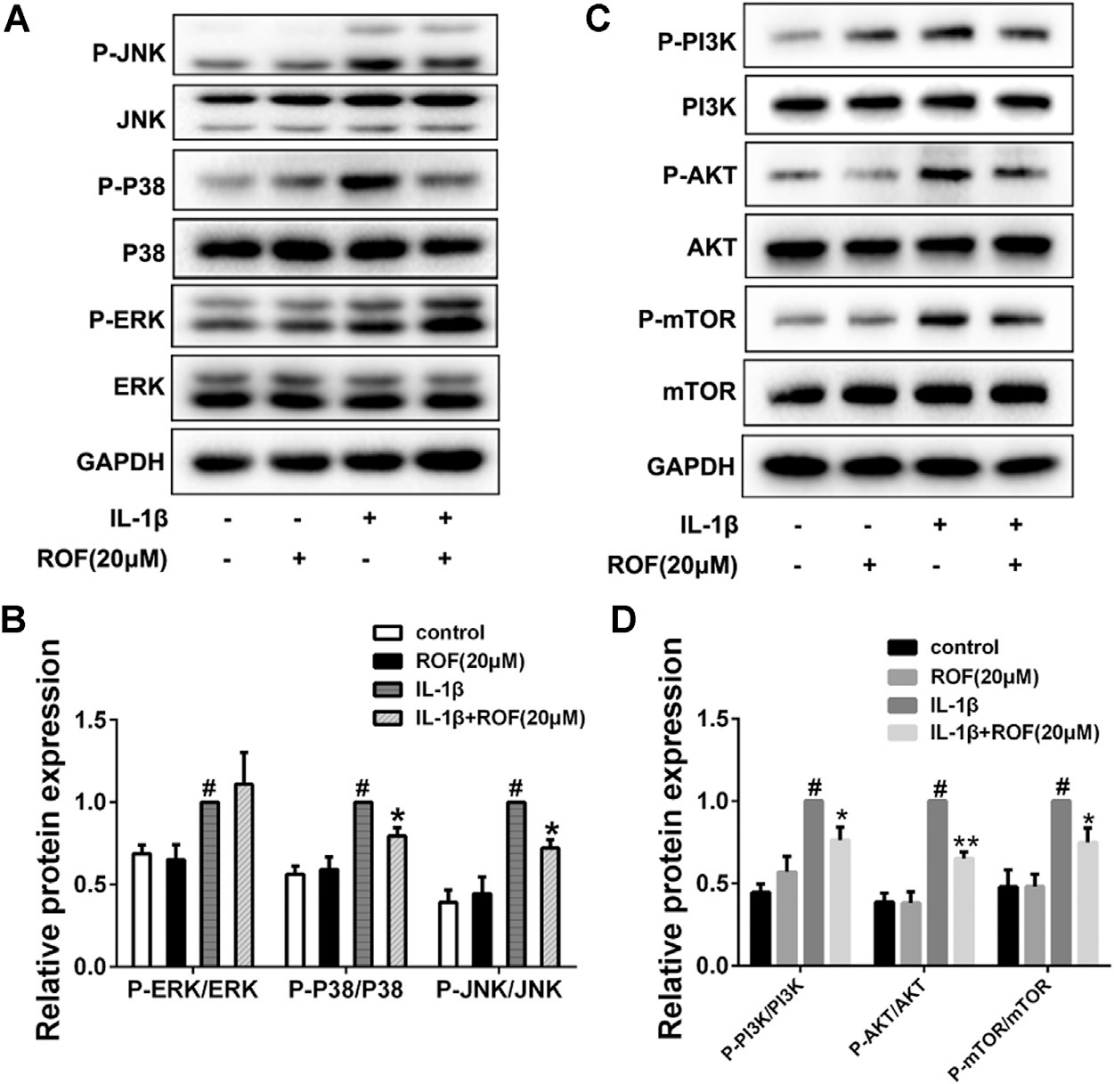
ROF blocks the activation of P38/JNK and PI3K/AKT/mTOR pathways induced by IL-1β in chondrocytes. Cells were exposed to L-1β (10 ng/ml) with or without 20 μM ROF for 30 min as above. **(A)** Western blots and **(B)** quantitative analysis of MAPK pathways in each group. **(C)** Western blots and **(D)** quantitative analysis of PI3K/AKT/mTOR pathways in each group. JNK, P38, ERK, PI3K, AKT, and mTOR were used as loading control (*n* = 3). #*p* < 0.05 vs. control group; *p < 0.05 and **p < 0.01 vs. IL-1β group.

MAPK and PI3K/AKT/mTOR pathways are highly associated with inflammatory reaction and autophagy in OA development. In this study, chondrocytes were first serum-starved overnight and then treated with IL-1β alone or in combination with 20 μM ROF for 30 min. As shown in Figure 5, ROF could dramatically block the phosphorylations of JNK, P38, PI3K, AKT. and mTOR induced by IL-1β in chondrocytes.

### Effects of Rhoifolin on Rat Osteoarthritis Model

**FIGURE 6:**
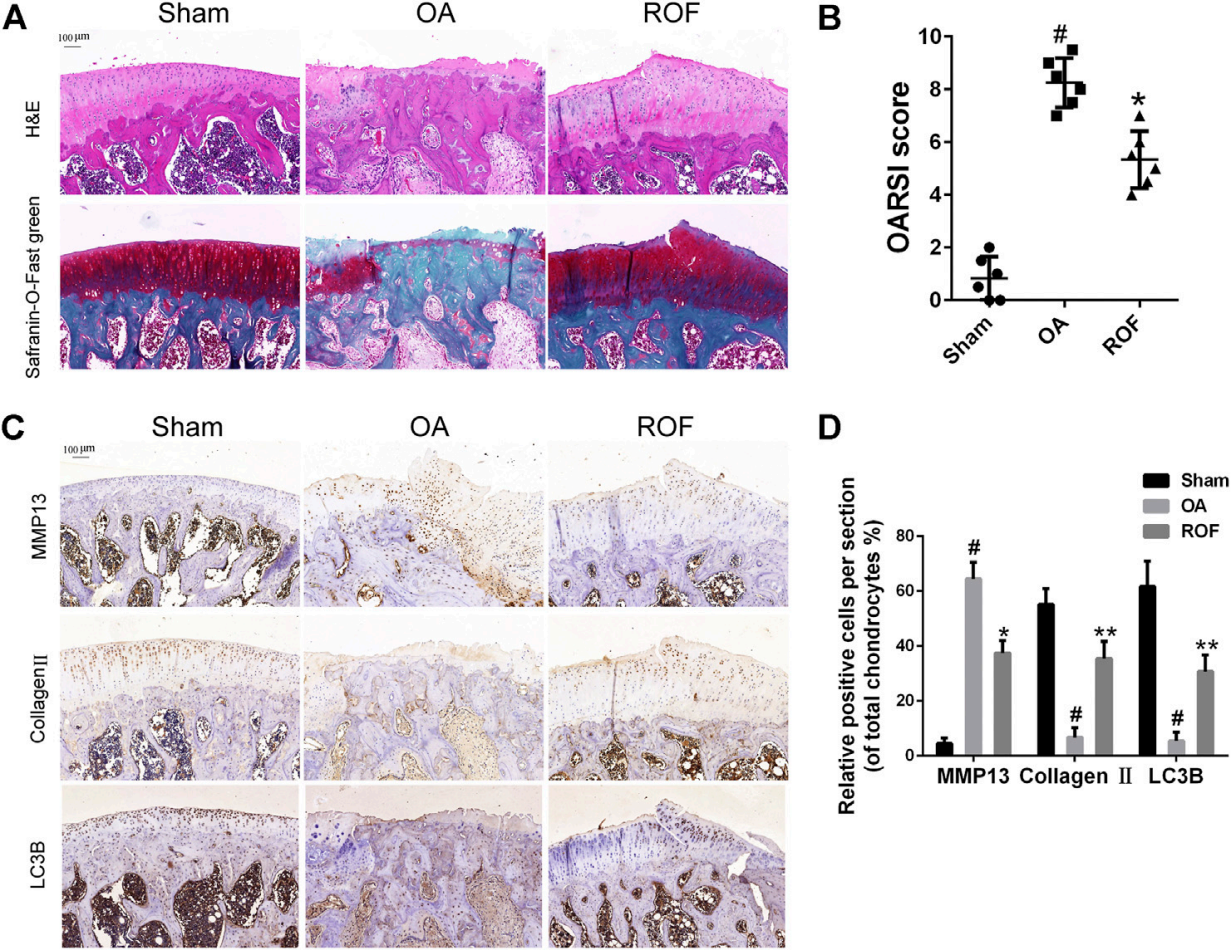
ROF alleviates cartilage damage in the rat OA model. **(A)** H&E and Safranin-O-Fast green staining of cartilage samples from three groups 8 weeks after surgery. **(B)** OARIS scores of three groups. **(C)** Immunohistochemical staining and **(D)** quantitative analysis of MMP13, collagen Ⅱ, and LC3B in the cartilage samples from three groups. The rate of positively stained chondrocytes in each section was calculated and quantitated from six rats of each group. #*p* < 0.05 vs. sham group; *p < 0.05 and **p < 0.01 vs. OA group.

To clarify the effects of ROF on rat OA *in vivo*, we built rat OA models by anterior cruciate ligament transaction and medial meniscus destabilization. All animals recovered with no infection or complications. ROF was injected into the knee joint weekly postsurgery. H&E and Safranin-O-Fast green staining were employed to access the histomorphology differences among samples from the three groups. As shown in Figure 6A, compared with normal structure of cartilage in the sham group, obvious cartilage damage, including surface erosion, disorganized sequence of chondrocytes, and loss of proteoglycan, was observed in the OA group. However, less cartilage lesion was observed in the ROF group. We further compared the OARSI scores in the three groups; results indicated that intra-articular injection of 20 μM ROF could markedly alleviate the OA progression (Figure 6B). In consistent with *in vitro* results, the immunohistochemistry staining showed that ROF could significantly increase collagen Ⅱ and LC3B and reduce MMP13 expression in rat OA cartilage (Figures 6C,D). All data indicated ROF could ameliorate OA progression *in vivo*.

## Discussion

Due to unsatisfactory therapeutic strategies available, osteoarthritis undoubtedly brings endless pain to the elderly (Robinson et al., 2016). In recent years, natural plant extract has attracted wide attention in OA therapy for its potential anti-inflammatory properties and mild side effects (Dragos et al., 2017). Rhoifolin (ROF), a flavanone first extracted from *Rhus succedanea*, has showed its anti-inflammatory potential in lipopolysaccharide-induced acute inflammation (Fang et al., 2020), Freund’s adjuvant–induced rheumatoid arthritis (Peng et al., 2020), and age-related inflammation (Lim et al., 2017). In this study, for the first time, we report the therapeutic effects of ROF in rat OA as well as the underlying mechanisms.

Local inflammatory responses and metabolic dysfunction are essential to the progression of OA. During the OA process, massive proinflammatory cytokines such as IL-1β and TNFα were secreted in the joint. These proteins can induce excessive production of inflammatory mediators and cartilage matrix–degrading enzymes (Dumond et al., 2004; Murphy and Nagase 2008). Previous study indicated that IL-1β at the dose of 10 ng/ml shared the maximum effects in rat chondrocytes (Wang, et al., 2018). Therefore, in our study, IL-1β treatment (10 ng/ml) was employed as a stimulus *in vitro*. We first confirmed ROF could significantly alleviate the IL-1β–induced upregulation of iNOS and COX-2. Moreover, our data revealed that ROF could reduce the IL-1β–induced production of MMP13 and ADAMTS5, thus contributing to the elevated level of collagen Ⅱ in chondrocytes. Taken together, we proved the anti-inflammatory and anti-cartilage degenerative effects of ROF on the IL-1β–treated rat chondrocytes.

Autophagy is considered as a protective process in normal cartilage (Carames et al., 2010). Enhancement of autophagy can delay the progression of OA via regulating intracellular metabolic activity (Luo et al., 2019). In our study, reduced levels of autophagy were observed in IL-1β–treated rat chondrocytes compared to normal ones. However, administration of ROF could partly reverse the IL-1β–induced autophagy downregulation. To further verify whether ROF exerts anti-inflammatory and anti-cartilage degradation effects via regulating autophagy, we used autophagy inhibitor 3-methyladenine (3-MA). It is interesting to see that ROF lost its protective properties when mixed with 3-MA. These results suggested that ROF ameliorated OA by regulating autophagy.

MAPK and PI3K/AKT/mTOR pathways are crucial to the onset and development of OA (Malemud 2017). Meanwhile, these two pathways are highly related to autophagy process (Yu et al., 2018). Previous study revealed inactivating MAPK signaling resulted in reduced cartilage and subchondral bone damage in OA (Zhou et al., 2019). Besides, inhibition of PI3K/AKT/mTOR promoted the autophagy and attenuated inflammation in OA (Xue et al., 2017). In our results, ROF could dramatically block the IL-1β–induced phosphorylations of P38/JNK and PI3K/AKT/mTOR pathways, indicating the underlying mechanisms. Since numerous natural compounds had been reported to block the IL-1β–induced activation of ERK in chondrocytes, it is interesting to see that ERK was not involved in the protective role of ROF in IL-1β–treated rat chondrocytes.

To clarify the therapeutic effects of ROF in OA, the *in vitro* study is far from enough. We next constructed the rat OA model and evaluated the protective properties of ROF *in vivo*. Results from the histological analysis indicated intra-articular injection of ROF could alleviate the cartilage damage. The *in vivo* data further confirmed the protective role of ROF in OA progression. However, the direct target of ROF in OA therapy is still unknown. Furthermore, the most suitable dose of ROF for *in vivo* application remains unclear. Considering the results we have achieved, further work is needed.

## Conclusion

In summary, we are the first to report the anti-inflammatory, anti-cartilage degradation and autophagy promoting properties of ROF in OA. We further proved ROF functioned by regulating autophagy. Moreover, P38/JNK and PI3K/AKT/mTOR pathways were involved in this process. Our study would contribute to the knowledge of the therapeutic effects of ROF and provide new ideas for future OA treatment.

## Data Availability

The raw data supporting the conclusion of this article will be made available by the authors, without undue reservation.
